# Hyperthermia-Based Anti-Cancer Treatments

**DOI:** 10.3390/cancers13061240

**Published:** 2021-03-12

**Authors:** Johannes Crezee, Nicolaas A. P. Franken, Arlene L. Oei

**Affiliations:** 1 Department of Radiation Oncology, Cancer Center Amsterdam, Amsterdam UMC, University of Amsterdam, Meibergdreef 9, 1105AZ Amsterdam, The Netherlands; n.a.franken@amsterdamumc.nl (N.A.P.F.); a.l.oei@amsterdamumc.nl (A.L.O.); 2 Laboratory for Experimental Oncology and Radiobiology, Cancer Center Amsterdam, Amsterdam UMC, University of Amsterdam, Meibergdreef 9, 1105AZ Amsterdam, The Netherlands; 3 Center for Experimental Molecular Medicine, Amsterdam UMC, University of Amsterdam, Meibergdreef 9, 1105AZ Amsterdam, The Netherlands

Hyperthermia is an adjuvant local anti-cancer treatment using temperatures exceeding the physiologically optimal level, typically 40–43 °C for approximately one hour. Hyperthermia applied as radiosensitizer or chemosensitizer has shown great results in over four decades and is presently successfully applied in combination with radiotherapy or chemotherapy for treatment of many tumour types, including recurrent breast cancer, bladder cancer, cervical carcinoma, head & neck cancer, soft tissue sarcoma, and melanoma [1]. To further improve the effectiveness of hyperthermia delivery in the clinic, clinical trials and preclinical hyperthermia research aim at even better exploiting the pleiotropic effects of hyperthermia, and technical research is performed into better controlled and more effective forms of heat delivery. This special issue collects 17 papers (16 original research papers and 1 review) that highlight recent developments in this buoyant and clinically relevant research domain.

Hyperthermia affects cells and tissues in various ways [2]. It can directly alter the physical properties of cellular components, but it can also influence cellular responses. Hyperthermia affects multiple intracellular processes, like e.g., DNA repair pathways, as well as systemic immune responses. Furthermore, hyperthermia can target cancer cells in hypoxic and nutrient-deprived tumour areas where ionising radiation and chemotherapy are least effective. Hyperthermia can also modify factors that are essential for tumour survival and growth, such as the microenvironment, immune responses, vascularisation, and oxygen supply [3]. Thus, the effects of hyperthermia are multifactorial, as addressed in the contributions in this issue. Hyperthermia inhibits the homologous recombination (HR) DNA repair pathway by inducing proteasomal degradation of BRCA2 [4]. Van den Tempel et al. investigated the mechanisms driving hyperthermia-induced BRCA2 degradation, finding that BRCA2 degradation is evolutionarily conserved, BRCA2 stability is dependent on HSP90, ubiquitin might not be directly involved, and BRCA2 degradation might be modulated by oxidative stress and radical scavengers [5]. Mei et al. investigated the impact of temperature (37–42 °C), sequence and time interval (0 up to 4 h) for ionizing radiation combined with hyperthermia on different HPV-positive and HPV-negative cervical cancer cell lines, demonstrating that shorter time intervals were associated with more unrepaired DNA damage and more tumour cell kill, especially at higher temperatures [6]. Hyperthermia at 42 °C was also demonstrated to have a marked potential in inducing an immunes response in an in vivo mouse model of colon adenocarcinoma, where intravenous administration of a human CCL3 variant carrying a single amino acid substitution after mild local hyperthermia treatment not only induced significant tumour growth inhibition but also inhibited metastasis [7]. Hader et al. investigated the impact of the heating method on changes in the immune phenotype of tumour cells, comparing the effect of warm-water bath versus 2.45 GHz microwave heating on cell death, the release of HSP70, and the expression of immune checkpoint molecules (ICMs) on breast cancer cells. They observed release of HSP70 after hyperthermia at a range of temperatures and independently of the heating method, but microwave heating was more effective in cell killing, and microwave heating with and without radiotherapy increased subsequent HSP70 concentrations [8].

Presently commercially available hyperthermia devices are available that are capable of ensuring effective heat delivery in many tumour sites [9]. Further progress is ongoing to develop dedicated equipment for specific sites, improve tumour control or facilitate a more effective work flow during treatment. Reproducibility of applicator shape and position is needed for accurate planning of superficial hyperthermia treatments, Kok et al. present a fast reconstruction method for bendable superficial hyperthermia applicators suitable for routine clinical patient-specific treatment planning [10]. Schooneveldt et al. developed and evaluated an advanced fluid dynamics treatment planning model of cerebrospinal fluid (CSF) to predict the temperature distribution when using a dedicated hyperthermia device developed for treatment of paediatric brain tumours [11]. Magnetic resonance thermometry (MRT) is emerging as a clinical non-invasive 3D-temperature measurement method. Curto et al. found good MRT accuracy for MR-hyperthermia hybrid systems at five European institutes while heating a centric or eccentric target in anthropomorphic phantoms with pelvic and spine structures [12]. Establishing antenna phase and amplitude settings resulting in optimal tumour heating is challenging and time-consuming for modern multi-antenna systems, a contribution of Kuehne et al. presents an elegant and fast solution for establishing global optimality, automatically determining optimum application RF frequencies and time-multiplexed RF excitations for desired target regions, desired RF power deposition patterns and constraints [13]. Advanced applicator design includes the design and test of a novel 32-channel modular signal source for heating deep-seated tumours. The large number of coherent RF channels, wide frequency range, and accurate phase shift provided by this source form a sound basis for well controlled, Magnetic Resonance (MR) guided hyperthermia treatment delivery [14]. A step further is a Thermal magnetic resonance (ThermalMR) feasibility study using an integrated RF applicator accommodating radio frequency (RF)-induced temperature modulation, thermometry, anatomic and functional imaging using a 7.0-tesla whole-body MR scanner, and (nano)molecular probing, aiming at controlled release at the tumour site of therapeutics from thermoresponsive nanogels [15]. Encapsulating drugs in temperature-sensitive liposomes is a novel method to enhance the distribution and concentration of drugs in tumours. Besse et al. investigated effectiveness and tumour drug distribution after treatment with different doses of different liposomal doxorubicin formulations of in mice bearing a human fibrosarcoma. Results indicate that tumour drug distribution is important for effective treatment, and Thermodox combined with hyperthermia resulted in the highest tissue drug levels [16]. Photothermal tumour ablation can serve as an alternative to classic surgery in tumour treatment. Kim et al. numerically analysed optimal conditions for laser–tissue interactions in gold nanoparticle (GNP)-enhanced photothermal therapy resulting in good tumour control while preventing overheating and damage to surrounding normal tissues [17]. Yu et al. developed a novel hydroxyethyl starch based on nanoparticles loaded with doxorubicin and indocyanine green. This combination showed high photothermal efficiency and tended to accumulate inside tumours compared to other major organs in a H22-tumour-bearing mouse model [18].

Clinical application of hyperthermia as part of multi-modality treatments has been gaining popularity in recent years. The effectiveness of radiotherapy as well as chemotherapy can be enhanced substantially by hyperthermia, resulting in significantly improved tumour control and prolonged disease-free survival [1]. Moreover, hyperthermia does so without increasing radiation or chemo-related side-effects. An important issue in clinical hyperthermia is establishing its dose-effect relationship. Unsoeld et al. showed that the tumour temperature measured with non-invasive MR-thermometry was significantly higher for high-risk soft tissue sarcomas showing a pathologic response in the resection specimen after preoperative radio(chemo)therapy and locoregional hyperthermia treatment than in tumours without pathologic response [19]. Tumour size is considered one of the most important prognostic factors when treating unresectable breast cancer, but is so far reported very heterogeneously. Notter et al. propose and apply a novel and simple size classification into distinct prognostic groups (rClasses 0–IV) for evaluation of 201 patients with pre-irradiated locally recurrent breast cancer [20]. Oldenborg et al. retrospectively analysed the impact of radiotherapy technique and fractionation schedule of postoperative reirradiation combined with hyperthermia on treatment outcome and late toxicity for patients with recurrent breast cancer, with lower radiation fraction dose appearing less toxic [21]. Cytoreductive surgery (CRS) with hyperthermic intraperitoneal chemotherapy (HIPEC) is an effective and increasingly popular treatment option with curative intent. Helderman et al. reviewed the literature and found that the type of drug, drug concentrations, carrier solution, volume of the perfusate, temperature of the perfusate, duration of the treatment, the technique of delivery, and patient selection are relevant factors for efficacy of HIPEC [22].

Concluding, the 17 contributions in this special issue present a representative cross-section of state-of-the-art hyperthermia research and show successful present and future clinical hyperthermia applications. Interest in clinical hyperthermia treatment continues to rise, and this has led to a new special issue entitled “hyperthermia in cancer” which continues to accept new manuscripts until 30 April 2021 [23]. Ten original research and review papers have been included until now (status 9 March 2021). Again a range of relevant topics is covered, including technical design and innovations for improved hyperthermia treatment delivery [24,25,26,27,28], a novel small animal model for preclinical HIPEC research [29], as well as clinical application of mild hyperthermia, thermal ablation and HIPEC for bladder cancer, prostate cancer, hepatocellular carcinoma, and peritoneal surface malignancies [30,31,32,33].
